# Supplementary material for: Sepsis under pressure, intraoperative surgical site infection prevention practices among nurses in emergency surgical settings: A qualitative study
Source: PLoS One. 2026 Jan 8;21(1):e0340833. doi: 10.1371/journal.pone.0340833 (PMC12782374; doi:10.1371/journal.pone.0340833)
Supplement: Attachment — Submitted filename: Response to Reviewers.docx [file pone.0340833.s002.docx]

**Response to Reviewer(s)**

**Submission ID: [PONE-D-25-42877] - [EMID:c25ba633eee736ee]**

**Title of Manuscript : Sepsis under pressure, intraoperative surgical site infection prevention practices among nurses in emergency surgical settings: a qualitative study**

Dear Editor,

We are grateful for giving us the opportunity to revise our manuscript for resubmission. All

the concerns raised by the reviewer have been addressed accordingly.

Kindly find below our responses to the points raised by the reviewer. All the authors have

agreed to the revisions made in the manuscript for resubmission.

Yours sincerely,

Gwendolyn Patience Mensah (PhD)

(Corresponding Author)

| **No.** | **Reviewer’s Comments** | **Authors’ Response** | **Page Number(s)** |
| --- | --- | --- | --- |
|  | **JOURNAL REQUIREMENTS** |  |  |
| 1 | When submitting your revision, we need you to address these additional requirements.  1. Please ensure that your manuscript meets PLOS ONE's style requirements, including those for file naming. | The mansucript’s file names have been revised to meet PLOS ONE's style requirements. |  |
| 2 | 2. Your ethics statement should only appear in the Methods section of your manuscript. If your ethics statement is written in any section besides the Methods, please move it to the Methods section and delete it from any other section. Please ensure that your ethics statement is included in your manuscript, as the ethics statement entered into the online submission form will not be published alongside your manuscript. | The ethics statements have been moved to the methods section of the manuscript as recommended. | **9** |
| 3 | 3. We note that you have indicated that there are restrictions to data sharing for this study. PLOS only allows data to be available upon request if there are legal or ethical restrictions on sharing data publicly. For more information on unacceptable data access restrictions, please see http://journals.plos.org/plosone/s/data-availability#loc-unacceptable-data-access-restrictions.  Before we proceed with your manuscript, please address the following prompts:  a) If there are ethical or legal restrictions on sharing a de-identified data set, please explain them in detail (e.g., data contain potentially identifying or sensitive patient information, data are owned by a third-party organization, etc.) and who has imposed them (e.g., a Research Ethics Committee or Institutional Review Board, etc.). Please also provide contact information for a data access committee, ethics committee, or other institutional body to which data requests may be sent.  b) If there are no restrictions, please upload the minimal anonymized data set necessary to replicate your study findings to a stable, public repository and provide us with the relevant URLs, DOIs, or accession numbers. For a list of recommended repositories, please see  https://journals.plos.org/plosone/s/recommended-repositories. You also have the option of uploading the data as Supporting Information files, but we would recommend depositing data directly to a data repository if possible.  We will update your Data Availability statement on your behalf to reflect the information you provide | The relevant anonnymised data have been provided in the manuscript. | **Pages 10-21** |
| 4 | 4. If the reviewer comments include a recommendation to cite specific previously published works, please review and evaluate these publications to determine whether they are relevant and should be cited. There is no requirement to cite these works unless the editor has indicated otherwise. |  |  |
|  | **REVIEWER #1** |  |  |
| 5 | 1. The research design aligns with the study aim of understanding intraoperative surgical site infection (SSI) prevention practices among nurses in a tertiary hospital. The use of semi-structured interviews and thematic analysis is suitable for the exploratory nature of the work. | The authors are grateful for the feedback. |  |
| 6 | The data collection procedures are adequately described, and the inclusion of direct participant quotations supports the authenticity of the findings. However, the analytic process lacks sufficient transparency. There is minimal description of the coding framework, how the codes were developed, refined and integrated into the themes. Although NVivo software is mentioned, its role in data management or theme generation is not clearly explained. | The analytical processes used have been provided as recommended.  The table of themes, subthemes and codes have been provided to show how codes were developed, refined and integrated into the final themes. | 7 and 8 |
| 7 | The findings and conclusions are largely consistent, with the data supporting the main claims about infection prevention practices, supervision, and resource challenges. However there is overgeneralization —for example, “multidisciplinary collaboration significantly reduces SSIs” that extends beyond what qualitative data can demonstrate. | We are grateful for this feedback. The overgeneralizing statements have been revised and limited to the qualitative data in the study. | 3 |
| 8 | There should be explicit documentation of ethics approval (e.g., institutional review board reference number).  Overall, the manuscript requires greater methodological transparency and moderation of inferential claims to fully substantiate its conclusions. | The institutional review board reference number was provided in the first document under Declarations section (Ethical Approval and Consent to participate). It has now been moved to the methods section. | 9 |
| 9 | 2. The manuscript provides only a superficial description of the analytic process: It lists Braun & Clarke’s phases but does not show how themes were derived from codes. There are no sample codes, codebooks, or excerpts to illustrate analytic progression. The process of reflexivity and peer debriefing is mentioned but not demonstrated with examples. while the method is suitable, the reporting of its application lacks depth and transparency. | We appreciate this feedback. A more detailed description of how themes (and subthemes) were derived from codes have been provided as recommended.  The process of reflexivity and peer debriefing has also been detailed. | 7, 8 and 9  12,13 and 14 |
| 10 | 3. No details on inter-coder reliability or peer validation (e.g., whether another researcher cross-checked coding).  No clear evidence of data triangulation or how saturation was confirmed. There is Over-reliance on researcher interpretation without documented audit examples. | Additional details have been provided regarding peer validation, particularly how the other researchers cross-checked the preliminary coding.  We have added that saturation was confirmed by conducting 2 additional interviews beyond the point where participants’ feedback became repetitive, additional interviews were not yielding new insights to add up to the themes and subthemes. | 6, 8 and 9 |
| 11 | 4. The manuscript lacks frequency indicators for reporting prevalence without quantifying. There is no comparative analysis (e.g., by role, experience, or theatre vs ward) explored, which could have added analytic richness. | We are grateful for this feedback. As recommended, we will explore these comparative analysis in follow-up studies to enhance the analytic richness. |  |
| 12 | 5. The manuscript does not include a Data Availability Statement, nor does it indicate where the underlying qualitative data (e.g., anonymized transcripts, coding framework) are deposited. | The Data Availability Statement was provided in the manuscript (page 20) under the declarations section (Availability of data and materials). | 25 |
| 13 | 6. The manuscript is presented in an intelligible and well-structured manner overall. The English is clear though minor grammatical and stylistic issues should be addressed g. Some sentences are overly long or repetitive | We are grateful for the feedback. We have reduced the overly long or repetitive sentences as recommended. Grammatical errors have been corrected. | 23 |
